# The genome sequence of the narrow-cheeked clusterfly, *Pollenia angustigena *(Wainwright, 1940)

**DOI:** 10.12688/wellcomeopenres.19576.1

**Published:** 2023-06-22

**Authors:** Steven Falk, Olga Sivell, University of Oxford and Wytham Woods Genome Acquisition Lab, Darwin Tree of Life Barcoding collective, Wellcome Sanger Institute Tree of Life programme, Wellcome Sanger Institute Scientific Operations: DNA Pipelines collective, Tree of Life Core Informatics collective, Darwin Tree of Life Consortium

**Affiliations:** 1 Independent researcher, Kenilworth, England, UK; 2 Natural History Museum, London, England, UK

**Keywords:** Pollenia angustigena, narrow-cheeked clusterfly, genome sequence, chromosomal, Diptera

## Abstract

We present a genome assembly from an individual female *Pollenia angustigena* (the narrow-cheeked clusterfly; Arthropoda; Insecta; Diptera; Polleniidae). The genome sequence is 1370.5 megabases in span. Most of the assembly is scaffolded into 6 chromosomal pseudomolecules, including the X sex chromosome. The mitochondrial genome has also been assembled and is 21.01 kilobases in length. Gene annotation of this assembly on Ensembl identified 12,930 protein coding genes.

## Species taxonomy

Eukaryota; Metazoa; Eumetazoa; Bilateria; Protostomia; Ecdysozoa; Panarthropoda; Arthropoda; Mandibulata; Pancrustacea; Hexapoda; Insecta; Dicondylia; Pterygota; Neoptera; Endopterygota; Diptera; Brachycera; Muscomorpha; Eremoneura; Cyclorrhapha; Schizophora; Calyptratae; Oestroidea; Polleniidae; *Pollenia*; *Pollenia angustigena* (Wainwright, 1940) (NCBI:txid1266490).

## Background

*Pollenia angustigena (*Diptera, Polleniidae) is a medium-size (4.5–9.5 mm) black fly with a chequered dusting pattern on the abdomen and golden crinkly hairs on the thorax. Until recently it was placed within Calliphoridae, but the Polleninae subfamily was given family status following molecular research by Cerretti *et al.* (2019). The species was considered a synonym of *Pollenia rudis* (Fabricius, 1794), (e.g. in Van Emden (1954) it appears as *P. rudis* f. *angustigena*), but this was rectified by Rognes (1985).

This species is somewhat variable in colour and morphology (e.g. palpi are yellow to black, the basicosta is usually yellow but can be dark; the facial keel is usually sharp but is occasionally low and blunt). *Pollenia angustigena* can be confused with other *Pollenia* species, particularly *P. rudis* ( Draber-Mońko, 2004; Rognes, 1987; Rognes, 1991; Sivell, 2021). The characteristic golden hairs on the posterior and posteroventral surface of the middle and hind femora can occasionally be found in *P. rudis* (particularly in specimens from central Europe), while the middle tibia, usually with a single anterodorsal (ad) bristle, can sometimes possess 2 to 3 ad, as in *P. rudis* ( Rognes, 1987). Rarely the outer post-humeral bristle may be missing, as in *P. griseotomentosa* (Jacentkovský, 1944), however other characters should allow for a correct identification ( Rognes, 1987). The males can be reliably separated based on the shape of the distiphallus and hairs on the anteroventral surface of the hind tibia, which are reclined in *P. angustigena* and erect and long in *P. rudis* ( Rognes, 1991; Sivell, 2021). Also, the ground vestiture on the ventral side of the abdomen is reclined, and as dense as on the dorsal side, while in *P. rudis* the ventral vestiture is erect, and denser and finer than the dorsal vestiture. In females the shape of lateral sacs is distinctive ( Rognes, 1991).

*Pollenia* are parasitoids and/or predators of earthworms. The eggs are laid in small batches on the ground and hatched larvae move through soil in search of a host ( Rognes, 1991). The larvae (individually or in small groups) penetrate the worm, usually dorsally. They feed on it internally with their spiracles exposed to the outside. Third instar larva can also feed on the exterior of the worm and can move on to a different host if needed. The larvae pupate in the soil ( Keilin, 1915; Rognes, 1987; Yahnke & George, 1972).

The first instar larva of *P. angustigena* was described by Szpila (2003), the puparium was described by Rognes (1987); the second and third instars remain unknown.

*Pollenia angustigena* is closely related to *P. rudis* and *P. pediculata* Macquart, 1834, as recently shown by molecular phylogenetic analysis using the three molecular markers, COI, Ef-1α and CAD ( Szpila *et al.*, 2023).

*Pollenia angustigena* is common and widely distributed in Britain ( Sivell, 2021). The adults feed on flowers, pollinating the plants ( Cerretti *et al.*, 2019; Grzywacz *et al.*, 2012; Jewiss-Gaines *et al.*, 2012; Szpila, 2003). This species is found in variety of habitats including woodland, grasslands, quarries ( Draber-Mońko, 2004; Sivell, 2021). It overwinters as an adult in sheltered locations such as buildings, where it clusters, often in large numbers (hence the common name “cluster fly”), potentially causing a nuisance ( Cerretti *et al.*, 2019; Rognes, 1987; Sivell, 2021). On the wing from March to October, although recorded all year round (in winter mainly indoors) ( Sivell, 2021).

The high-quality genome of *Pollenia angustigena* was sequenced as part of the Darwin Tree of Life Project, a collaborative effort to sequence all named eukaryotic species in the Atlantic Archipelago of Britain and Ireland. Here we present a chromosomally complete genome sequence for *Pollenia angustigena*, based on one specimen from Wytham Woods.

## Genome sequence report

**Figure 1.**
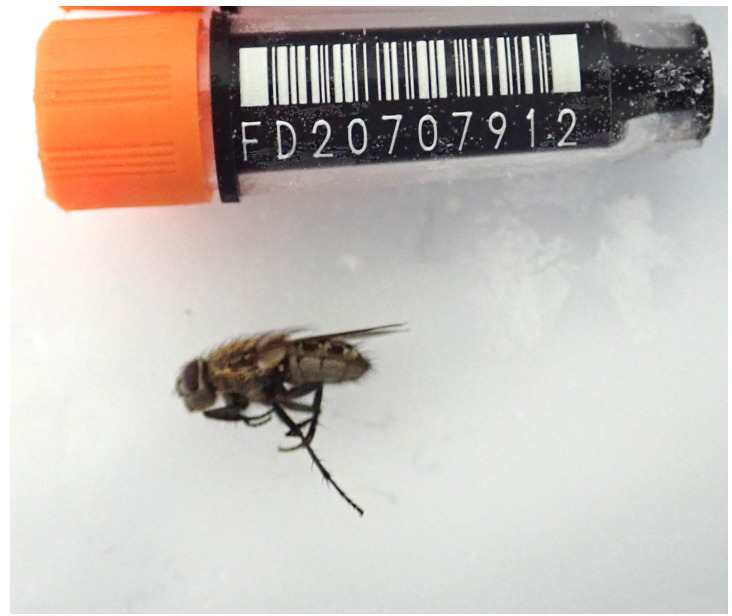
Photograph of the *Pollenia angustigena* (idPolAngu1) specimen used for genome sequencing.

The genome was sequenced from one female *Pollenia angustigena* ( Figure 1) collected from Wytham Woods, Oxfordshire, UK (51.77, –1.33). A total of 29-fold coverage in Pacific Biosciences single-molecule HiFi long reads and 28-fold coverage in 10X Genomics read clouds were generated. Primary assembly contigs were scaffolded with chromosome conformation Hi-C data. Manual assembly curation corrected 56 missing joins or mis-joins and removed 6 haplotypic duplications, reducing the assembly length by 0.35% and the scaffold number by 50.82%, and increasing the scaffold N50 by 17.67%.

**Figure 2.**
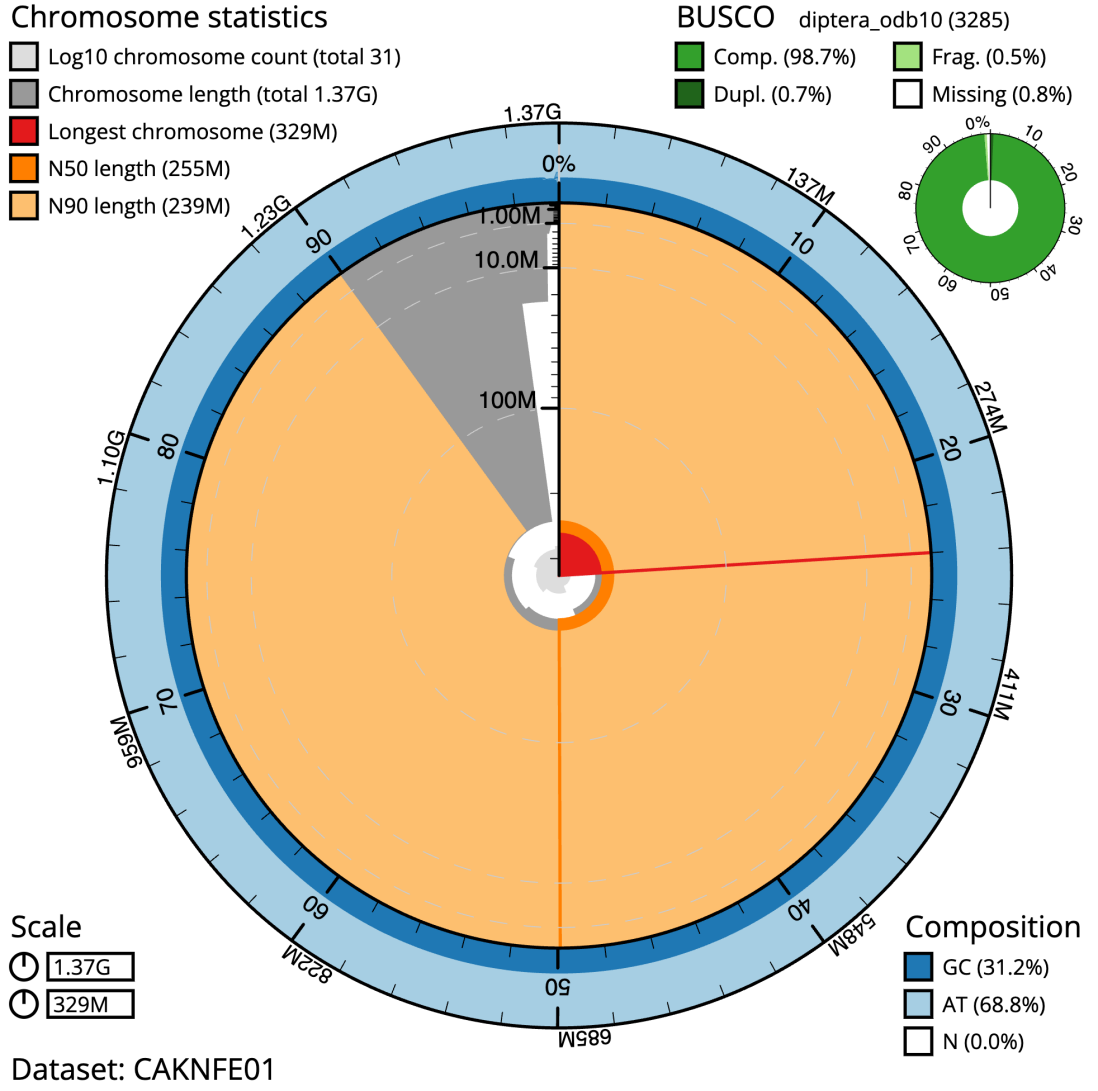
Genome assembly of *Pollenia angustigena*, idPolAngu1.1: metrics. The BlobToolKit Snailplot shows N50 metrics and BUSCO gene completeness. The main plot is divided into 1,000 size-ordered bins around the circumference with each bin representing 0.1% of the 1,370,512,213 bp assembly. The distribution of scaffold lengths is shown in dark grey with the plot radius scaled to the longest scaffold present in the assembly (329,103,898 bp, shown in red). Orange and pale-orange arcs show the N50 and N90 scaffold lengths (255,371,252 and 238,882,577 bp), respectively. The pale grey spiral shows the cumulative scaffold count on a log scale with white scale lines showing successive orders of magnitude. The blue and pale-blue area around the outside of the plot shows the distribution of GC, AT and N percentages in the same bins as the inner plot. A summary of complete, fragmented, duplicated and missing BUSCO genes in the diptera_odb10 set is shown in the top right. An interactive version of this figure is available at https://blobtoolkit.genomehubs.org/view/Pollenia angustigena/dataset/CAKNFE01/snail.

The final assembly has a total length of 1,370.5 Mb in 30 sequence scaffolds with a scaffold N50 of 255.4 Mb ( Table 1). Most (99.96%) of the assembly sequence was assigned to 6 chromosomal-level scaffolds, representing 5 autosomes and the X sex chromosome. Chromosome-scale scaffolds confirmed by the Hi-C data are named in order of size ( Figure 2– Figure 5; Table 2). While not fully phased, the assembly deposited is of one haplotype. Contigs corresponding to the second haplotype have also been deposited. The mitochondrial genome was also assembled and can be found as a contig within the multifasta file of the genome submission.

**Table 1.** Genome data for *Pollenia angustigena*, idPolAngu1.1.

Project accession data		
Assembly identifier	idPolAngu1.1	
Species	*Pollenia angustigena*	
Specimen	idPolAngu1	
NCBI taxonomy ID	1266490	
BioProject	PRJEB48114	
BioSample ID	SAMEA7746597	
Isolate information	idPolAngu1: thorax (DNA sequencing), head (Hi-C scaffolding), abdomen (RNA sequencing)	
Assembly metrics *		*Benchmark*
Consensus quality (QV)	51.7	*≥ 50*
*k*-mer completeness	99.97	*≥ 95%*
BUSCO **	C:98.7%[S:98.1%,D:0.7%], F:0.5%,M:0.8%,n:3,285	*C ≥ 95%*
Percentage of assembly mapped to chromosomes	99.96%	*≥ 95%*
Sex chromosomes	X chromosome	*localised homologous * *pairs*
Organelles	Mitochondrial genome assembled	*complete single alleles*
Raw data accessions		
PacificBiosciences SEQUEL II	ERR7123975, ERR7123976	
10X Genomics Illumina	ERR7113562–ERR7113565	
Hi-C Illumina	ERR7113561	
PolyA RNA-Seq Illumina	ERR10123660	
Genome assembly		
Assembly accession	GCA_930367215.1	
*Accession of alternate haplotype*	GCA_930374645.1	
Span (Mb)	1,370.5	
Number of contigs	142	
Contig N50 length (Mb)	21.0	
Number of scaffolds	30	
Scaffold N50 length (Mb)	255.4	
Longest scaffold (Mb)	329.1	
Genome annotation		
Number of protein-coding genes	12,930	
Number of non-coding genes	2,232	
Number of gene transcripts	20,301	

* Assembly metric benchmarks are adapted from column VGP-2020 of “Table 1: Proposed standards and metrics for defining genome assembly quality” from ( Rhie *et al.*, 2021). ** BUSCO scores based on the diptera_odb10 BUSCO set using v5.3.2. C = complete [S = single copy, D = duplicated], F = fragmented, M = missing, n = number of orthologues in comparison. A full set of BUSCO scores is available at https://blobtoolkit.genomehubs.org/view/Pollenia angustigena/dataset/CAKNFE01/busco.

**Figure 3.**
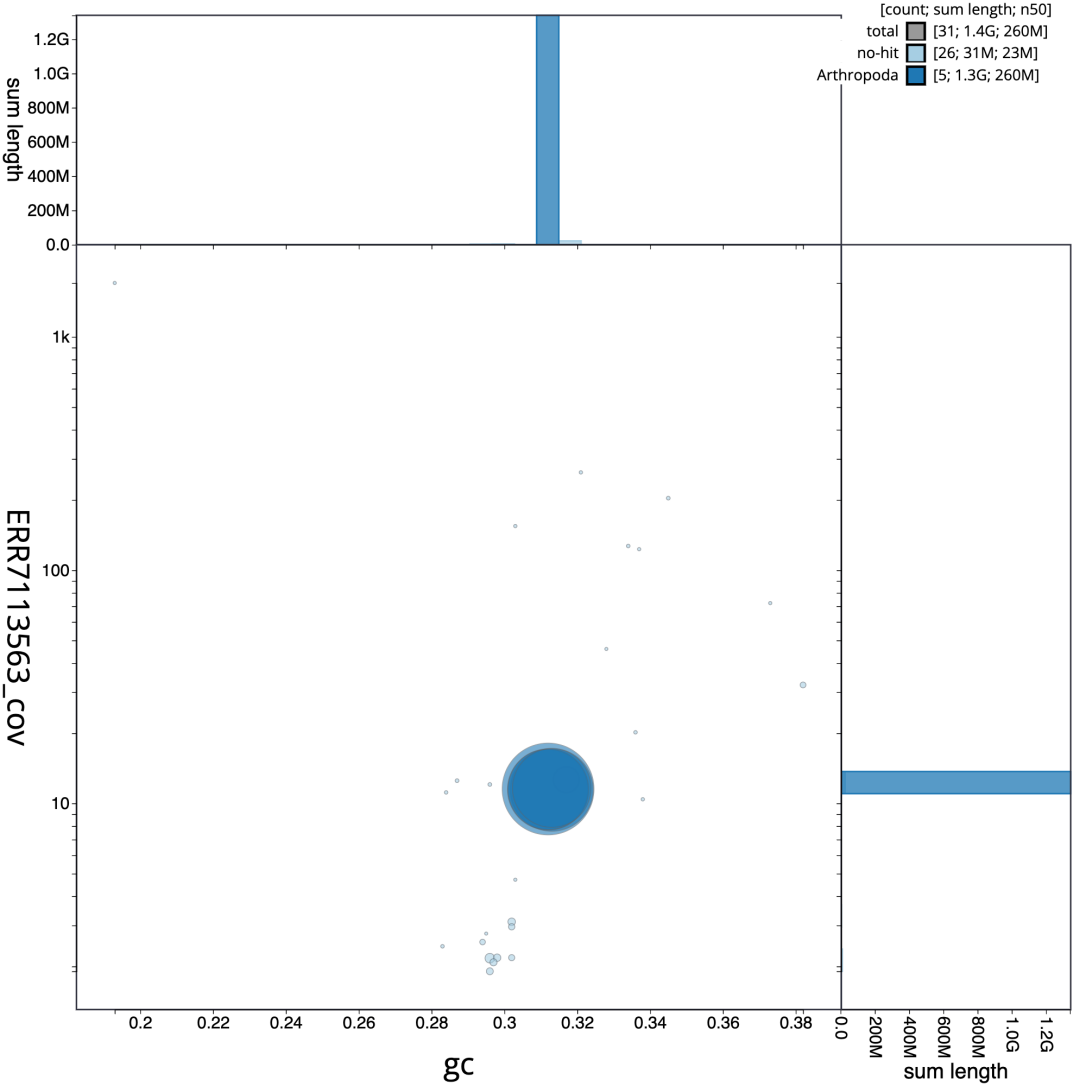
Genome assembly of *Pollenia angustigena*, idPolAngu1.1: BlobToolKit GC-coverage plot. Scaffolds are coloured by phylum. Circles are sized in proportion to scaffold length. Histograms show the distribution of scaffold length sum along each axis. An interactive version of this figure is available at https://blobtoolkit.genomehubs.org/view/Pollenia angustigena/dataset/CAKNFE01/blob.

**Figure 4.**
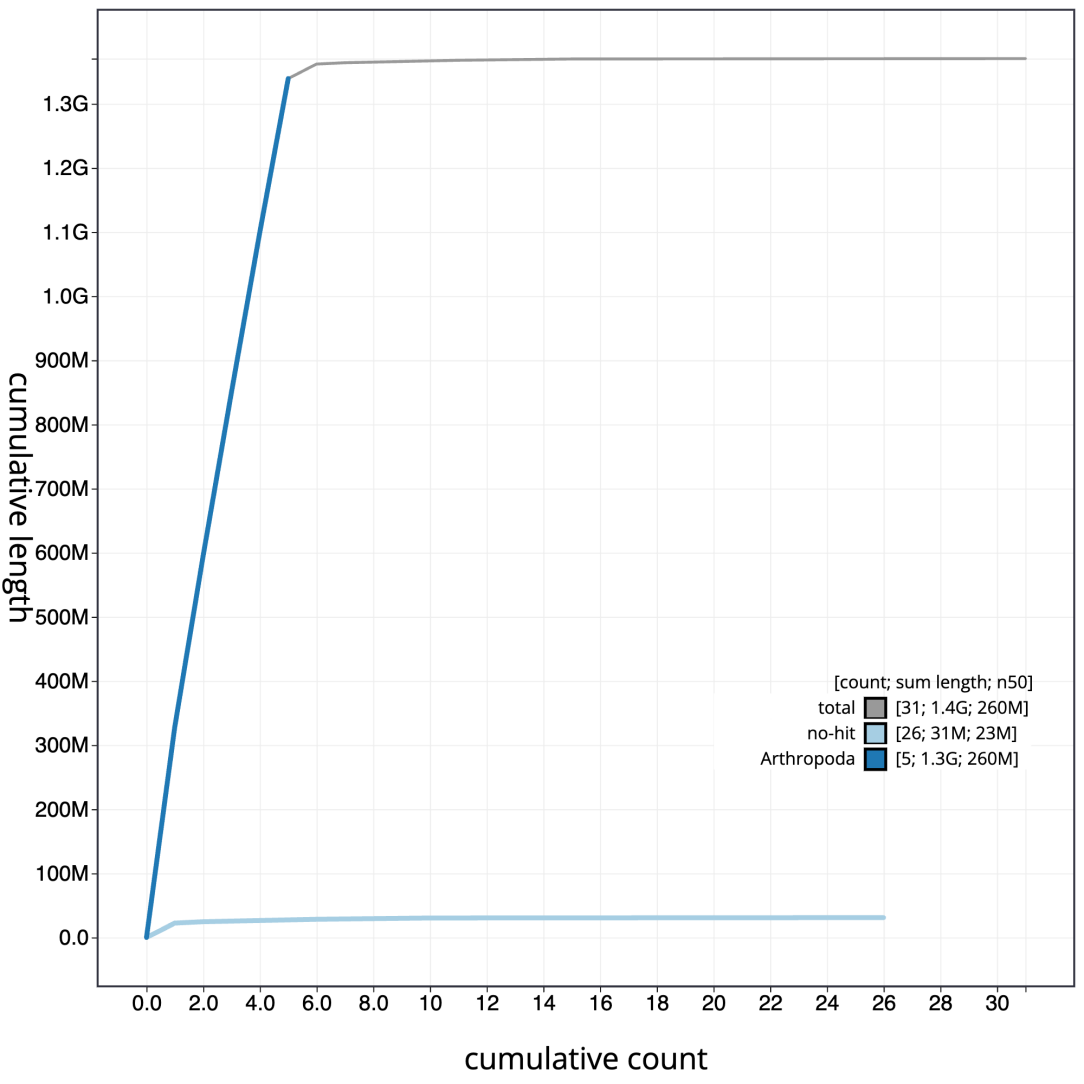
Genome assembly of *Pollenia angustigena*, idPolAngu1.1: BlobToolKit cumulative sequence plot. The grey line shows cumulative length for all scaffolds. Coloured lines show cumulative lengths of scaffolds assigned to each phylum using the buscogenes taxrule. An interactive version of this figure is available at https://blobtoolkit.genomehubs.org/view/Pollenia angustigena/dataset/CAKNFE01/cumulative.

**Figure 5.**
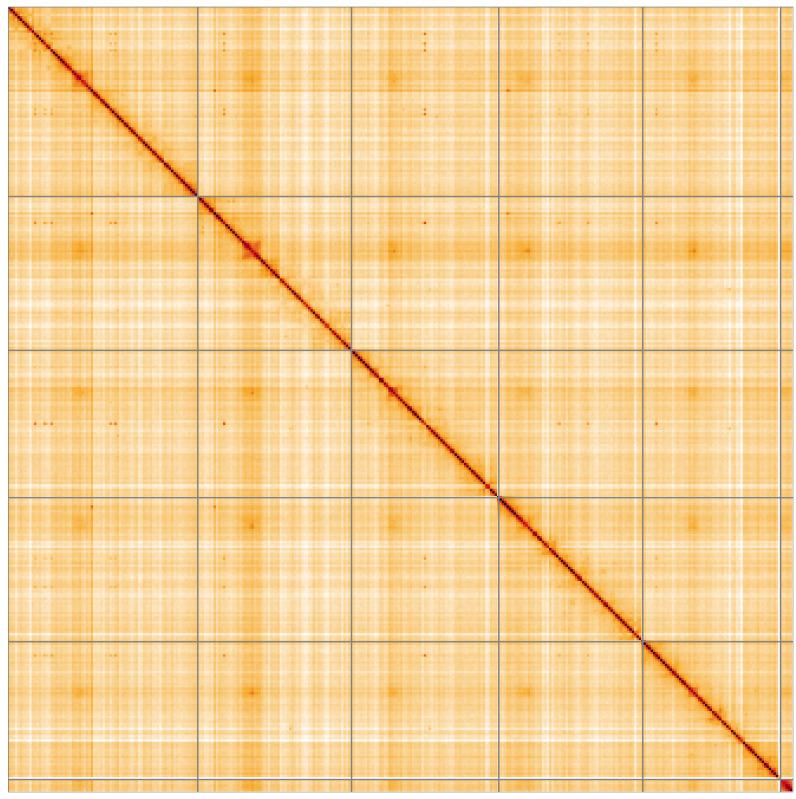
Genome assembly of *Pollenia angustigena*, idPolAngu1.1: Hi-C contact map of the idPolAngu1.1 assembly, visualised using HiGlass. Chromosomes are shown in order of size from left to right and top to bottom. An interactive version of this figure may be viewed at https://genome-note-higlass.tol.sanger.ac.uk/l/?d=LGCnoUK1QACjBH2Tiifsmg.

**Table 2.** Chromosomal pseudomolecules in the genome assembly of *Pollenia angustigena*, idPolAngu1.

INSDC accession	Chromosome	Length (Mb)	GC%
OV884056.1	1	329.1	31.0
OV884057.1	2	266.48	31.5
OV884058.1	3	255.37	31.0
OV884059.1	4	249.62	31.0
OV884060.1	5	238.88	31.5
OV884061.1	X	22.52	31.5
OV884062.1	MT	0.02	20.0

The estimated Quality Value (QV) of the final assembly is 51.7 with *k*-mer completeness of 99.97%, and the assembly has a BUSCO v5.3.2 completeness of 98.7% (single = 98.1%, duplicated = 0.7%), using the diptera_odb10 reference set ( *n* = 3,285).

Metadata for specimens, spectral estimates, sequencing runs, contaminants and pre-curation assembly statistics can be found at https://links.tol.sanger.ac.uk/species/1266490.

## Genome annotation report

The *Pollenia angustigena* genome assembly (GCA_930367215.1) was annotated using the Ensembl rapid annotation pipeline ( Table 1; https://rapid.ensembl.org/Pollenia_angustigena_GCA_930367215.1/Info/Index). The resulting annotation includes 20,301 transcribed mRNAs from 12,930 protein-coding and 2,232 non-coding genes.

## Methods

### Sample acquisition and nucleic acid extraction

A female *Pollenia angustigena* (idPolAngu1) was collected from Wytham Woods, Oxfordshire (biological vice-county Berkshire), UK (latitude 51.77, longitude –1.33) on 2020-08-04 by netting. The specimen was collected and identified by Steven Falk (independent researcher) and was then snap-frozen on dry ice.

DNA was extracted at the Tree of Life laboratory, Wellcome Sanger Institute (WSI). The idPolAngu1 sample was weighed and dissected on dry ice with tissue set aside for Hi-C sequencing. Thorax tissue was disrupted using a Nippi Powermasher fitted with a BioMasher pestle. High molecular weight (HMW) DNA was extracted using the Qiagen MagAttract HMW DNA extraction kit. Low molecular weight DNA was removed from a 20 ng aliquot of extracted DNA using the 0.8X AMpure XP purification kit prior to 10X Chromium sequencing; a minimum of 50 ng DNA was submitted for 10X sequencing. HMW DNA was sheared into an average fragment size of 12–20 kb in a Megaruptor 3 system with speed setting 30. Sheared DNA was purified by solid-phase reversible immobilisation using AMPure PB beads with a 1.8X ratio of beads to sample to remove the shorter fragments and concentrate the DNA sample. The concentration of the sheared and purified DNA was assessed using a Nanodrop spectrophotometer and Qubit Fluorometer and Qubit dsDNA High Sensitivity Assay kit. Fragment size distribution was evaluated by running the sample on the FemtoPulse system.

RNA was extracted from abdomen tissue of idPolAngu1 in the Tree of Life Laboratory at the WSI using TRIzol, according to the manufacturer’s instructions. RNA was then eluted in 50 μl RNAse-free water and its concentration assessed using a Nanodrop spectrophotometer and Qubit Fluorometer using the Qubit RNA Broad-Range (BR) Assay kit. Analysis of the integrity of the RNA was done using Agilent RNA 6000 Pico Kit and Eukaryotic Total RNA assay.

### Sequencing

Pacific Biosciences HiFi circular consensus and 10X Genomics read cloud DNA sequencing libraries were constructed according to the manufacturers’ instructions. Poly(A) RNA-Seq libraries were constructed using the NEB Ultra II RNA Library Prep kit. DNA and RNA sequencing were performed by the Scientific Operations core at the WSI on Pacific Biosciences SEQUEL II (HiFi), Illumina NovaSeq 6000 (RNA-Seq and 10X) instruments. Hi-C data were also generated from head tissue of idPolAngu1 using the Arimav2 kit and sequenced on the Illumina NovaSeq 6000 instrument.

### Genome assembly, curation and evaluation

Assembly was carried out with Hifiasm ( Cheng *et al.*, 2021) and haplotypic duplication was identified and removed with purge_dups ( Guan *et al.*, 2020). One round of polishing was performed by aligning 10X Genomics read data to the assembly with Long Ranger ALIGN, calling variants with FreeBayes ( Garrison & Marth, 2012). The assembly was then scaffolded with Hi-C data ( Rao *et al.*, 2014) using SALSA2 ( Ghurye *et al.*, 2019). The assembly was checked for contamination and corrected as described previously ( Howe *et al.*, 2021). Manual curation was performed using HiGlass ( Kerpedjiev *et al.*, 2018) and Pretext ( Harry, 2022). The mitochondrial genome was assembled using MitoHiFi ( Uliano-Silva *et al.*, 2022), which runs MitoFinder ( Allio *et al.*, 2020) or MITOS ( Bernt *et al.*, 2013) and uses these annotations to select the final mitochondrial contig and to ensure the general quality of the sequence.

A Hi-C map for the final assembly was produced using bwa-mem2 ( Vasimuddin *et al.*, 2019) in the Cooler file format ( Abdennur & Mirny, 2020). To assess the assembly metrics, the *k*-mer completeness and QV consensus quality values were calculated in Merqury ( Rhie *et al.*, 2020). This work was done using Nextflow ( Di Tommaso *et al.*, 2017) DSL2 pipelines “sanger-tol/readmapping” ( Surana *et al.*, 2023a) and “sanger-tol/genomenote” ( Surana *et al.*, 2023b). The genome was analysed within the BlobToolKit environment ( Challis *et al.*, 2020) and BUSCO scores ( Manni *et al.*, 2021; Simão *et al.*, 2015) were calculated.

Table 3 contains a list of relevant software tool versions and sources.

**Table 3.** Software tools: versions and sources.

Software tool	Version	Source
BlobToolKit	4.1.5	https://github.com/blobtoolkit/blobtoolkit
BUSCO	5.3.2	https://gitlab.com/ezlab/busco
FreeBayes	1.3.1-17- gaa2ace8	https://github.com/freebayes/freebayes
Hifiasm	0.15.3	https://github.com/chhylp123/hifiasm
HiGlass	1.11.6	https://github.com/higlass/higlass
Long Ranger ALIGN	2.2.2	https://support.10xgenomics.com/genome-exome/software/pipelines/latest/ advanced/other-pipelines
Merqury	MerquryFK	https://github.com/thegenemyers/MERQURY.FK
MitoHiFi	2	https://github.com/marcelauliano/MitoHiFi
PretextView	0.2	https://github.com/wtsi-hpag/PretextView
purge_dups	1.2.3	https://github.com/dfguan/purge_dups
SALSA	2.2	https://github.com/salsa-rs/salsa
sanger-tol/ genomenote	v1.0	https://github.com/sanger-tol/genomenote
sanger-tol/ readmapping	1.1.0	https://github.com/sanger-tol/readmapping/tree/1.1.0

### Genome annotation

The Ensembl gene annotation system ( Aken *et al.*, 2016) was used to generate annotation for the *Pollenia angustigena* assembly (GCA_930367215.1). Annotation was created primarily through alignment of transcriptomic data to the genome, with gap filling via protein-to-genome alignments of a select set of proteins from UniProt ( UniProt Consortium, 2019).

### Wellcome Sanger Institute – Legal and Governance

The materials that have contributed to this genome note have been supplied by a Darwin Tree of Life Partner. The submission of materials by a Darwin Tree of Life Partner is subject to the **‘Darwin Tree of Life Project Sampling Code of Practice’**, which can be found in full on the Darwin Tree of Life website here. By agreeing with and signing up to the Sampling Code of Practice, the Darwin Tree of Life Partner agrees they will meet the legal and ethical requirements and standards set out within this document in respect of all samples acquired for, and supplied to, the Darwin Tree of Life Project.

Further, the Wellcome Sanger Institute employs a process whereby due diligence is carried out proportionate to the nature of the materials themselves, and the circumstances under which they have been/are to be collected and provided for use. The purpose of this is to address and mitigate any potential legal and/or ethical implications of receipt and use of the materials as part of the research project, and to ensure that in doing so we align with best practice wherever possible. The overarching areas of consideration are:

- Ethical review of provenance and sourcing of the material
- Legality of collection, transfer and use (national and international)

Each transfer of samples is further undertaken according to a Research Collaboration Agreement or Material Transfer Agreement entered into by the Darwin Tree of Life Partner, Genome Research Limited (operating as the Wellcome Sanger Institute), and in some circumstances other Darwin Tree of Life collaborators.

## Data Availability

European Nucleotide Archive: *Pollenia angustigena* (narrow-cheeked clusterfly). Accession number PRJEB48114; https://identifiers.org/ena.embl/PRJEB48114. ( Wellcome Sanger Institute, 2022) The genome sequence is released openly for reuse. The *Pollenia angustigena* genome sequencing initiative is part of the Darwin Tree of Life (DToL) project. All raw sequence data and the assembly have been deposited in INSDC databases. Raw data and assembly accession identifiers are reported in Table 1.

## Author information

Members of the University of Oxford and Wytham Woods Genome Acquisition Lab are listed here: https://doi.org/10.5281/zenodo.4789928.

Members of the Darwin Tree of Life Barcoding collective are listed here: https://doi.org/10.5281/zenodo.4893703.

Members of the Wellcome Sanger Institute Tree of Life programme are listed here: https://doi.org/10.5281/zenodo.4783585.

Members of Wellcome Sanger Institute Scientific Operations: DNA Pipelines collective are listed here: https://doi.org/10.5281/zenodo.4790455.

Members of the Tree of Life Core Informatics collective are listed here: https://doi.org/10.5281/zenodo.5013541.

Members of the Darwin Tree of Life Consortium are listed here: https://doi.org/10.5281/zenodo.4783558.
