# Supplementary material for: The genome sequence of the narrow-cheeked clusterfly, Pollenia angustigena (Wainwright, 1940)
Source: Wellcome Open Res. 2023 Jun 22;8:270. [Version 1] doi: 10.12688/wellcomeopenres.19576.1 (PMC11437293; doi:10.12688/wellcomeopenres.19576.1)
Supplement: Peer review history

# Reviewer report: Reviewer response for version 1

**DOI:** 10.21956/wellcomeopenres.21686.r95987

**Published:** 2024-09-27

**Authors:** Alessandro Vezzi

**Affiliations:** 1 Department of Biology, University of Padova, Padova, Italy

*Pollenia angustigena* is common and widespread in the UK. This species is found in a variety of habitats and overwinters as an adult in sheltered places such as buildings, potentially causing a nuisance. Also, adults pollinate plants by feeding on flowers.

 Given its wide distribution in Britain, and its ecological importance as a pollinator, it seems obvious that the genome of this species should be sequenced, assembled and annotated by the Darwin Tree of Life project.

 Thanks to the sequencing effort, which involved the use of Pacific Biosciences single-molecule HiFi long reads and 10X Genomics read clouds, plus chromosome conformation Hi-C data, most of the assembly sequence was assigned to 6 chromosomal-level scaffolds, representing 5 autosomes and the X sex chromosome. The assembled genome is therefore of high quality.

 The genome annotation mainly involved the use of transcriptomic data aligned to the genome, which, being of high quality, certainly enabled easy alignment and correct reconstruction of the gene structure.

 I would like to suggest some improvements in some details of the experimental methods section, as

 1) The 0.8X **AMPure** XP purification kit does not exist. By 0.8X we mean the ratio of the volume of XP beads to the volume of sample to be purified.

 2) It is not clear how many ng of extracted DNA were used for 10X sequencing. In fact, initially it is written that "a 20 ng aliquot of extracted DNA" was purified before 10X Chromium sequencing. But then a minimum of 50 ng DNA was submitted for 10X sequencing. If I checked the sequencing runs correctly, you are talking about the same DNA sequenced with the same platform, so I am a little confused by the different ng used.

**Two final points I noticed in the article:**

 1) The reference ‘Jacentkovský,

 1944' is not linked to any article.

 2) Elongation factor-1α must be written **EF-1α**.

Are sufficient details of methods and materials provided to allow replication by others?

Partly

Is the rationale for creating the dataset(s) clearly described?

Yes

Are the datasets clearly presented in a useable and accessible format?

Yes

Are the protocols appropriate and is the work technically sound?

Yes

Reviewer Expertise:

Genomics and metagenomics, sequencing tecnologies.

I confirm that I have read this submission and believe that I have an appropriate level of expertise to confirm that it is of an acceptable scientific standard.

# Reviewer report: Reviewer response for version 1

**DOI:** 10.21956/wellcomeopenres.21686.r66119

**Published:** 2023-09-11

**Authors:** Shigeyuki Koshikawa

**Affiliations:** 1 Graduate School of Environmental Science, Faculty of Environmental Earth Science, Hokkaido University, Sapporo, Japan

This article reports a high-quality genome sequence of the narrow-cheeked clusterfly, *Pollenia angustigena*. The taxonomic position and ecology of this species are briefly presented. The methods used to obtain the genome sequence are explained in an appropriate amount of detail. I believe that this report will contribute to our understanding of local biodiversity and the biology of insects in general.

Are sufficient details of methods and materials provided to allow replication by others?

Yes

Is the rationale for creating the dataset(s) clearly described?

Yes

Are the datasets clearly presented in a useable and accessible format?

Yes

Are the protocols appropriate and is the work technically sound?

Yes

Reviewer Expertise:

Evolutionary developmental biology of insects

I confirm that I have read this submission and believe that I have an appropriate level of expertise to confirm that it is of an acceptable scientific standard.
